# Individual differences in brain aging: heterogeneity in cortico-hippocampal but not caudate atrophy rates

**DOI:** 10.1093/cercor/bhac400

**Published:** 2022-10-05

**Authors:** Lars Nyberg, Micael Andersson, Anders Lundquist, William F C Baaré, David Bartrés-Faz, Lars Bertram, Carl-Johan Boraxbekk, Andreas M Brandmaier, Naiara Demnitz, Christian A Drevon, Sandra Duezel, Klaus P Ebmeier, Paolo Ghisletta, Richard Henson, Daria E A Jensen, Rogier A Kievit, Ethan Knights, Simone Kühn, Ulman Lindenberger, Anna Plachti, Sara Pudas, James M Roe, Kathrine Skak Madsen, Cristina Solé-Padullés, Yasmine Sommerer, Sana Suri, Enikő Zsoldos, Anders M Fjell, Kristine B Walhovd

**Affiliations:** Department of Radiation Sciences (Radiology), Umeå University, 901 87 Umeå, Sweden; Department of Integrative Medical Biology, Umeå University, 901 87 Umeå, Sweden; Umeå Center for Functional Brain Imaging, Umeå University, 901 87 Umeå, Sweden; Center for Lifespan Changes in Brain and Cognition, University of Oslo, Department of Psychology, University of Oslo, 0373 Oslo, Norway; Department of Integrative Medical Biology, Umeå University, 901 87 Umeå, Sweden; Umeå Center for Functional Brain Imaging, Umeå University, 901 87 Umeå, Sweden; Umeå Center for Functional Brain Imaging, Umeå University, 901 87 Umeå, Sweden; Department of Statistics, USBE, Umeå University, Umeå S-90187, Sweden; Danish Research Centre for Magnetic Resonance, Centre for Functional and Diagnostic Imaging and Research, Copenhagen University Hospital - Amager and Hvidovre, 2650 Copenhagen, Denmark; Department of Medicine, Faculty of Medicine and Health Sciences, Institut de Neurociències, Universitat de Barcelona, and Institut d’Investigacions Biomèdiques August Pi I Sunyer (IDIBAPS), 08036 Barcelona, Spain; Center for Lifespan Changes in Brain and Cognition, University of Oslo, Department of Psychology, University of Oslo, 0373 Oslo, Norway; Lübeck Interdisciplinary Platform for Genome Analytics (LIGA), University of Lübeck, 23562 Lübeck, Germany; Department of Radiation Sciences (Radiology), Umeå University, 901 87 Umeå, Sweden; Umeå Center for Functional Brain Imaging, Umeå University, 901 87 Umeå, Sweden; Danish Research Centre for Magnetic Resonance, Centre for Functional and Diagnostic Imaging and Research, Copenhagen University Hospital - Amager and Hvidovre, 2650 Copenhagen, Denmark; Faculty of Medical and Health Sciences, Institute for Clinical Medicine, University of Copenhagen, 2400 Copenhagen, Denmark; Department of Neurology, Institute of Sports Medicine Copenhagen (ISMC), Copenhagen University Hospital - Bispebjerg and Frederiksberg, 2400 Copenhagen, Denmark; Center for Lifespan Psychology, Max Planck Institute for Human Development, 14195 Berlin, Germany; MSB Medical School Berlin, 14197 Berlin, Germany; Max Plank UCL Centre for Computational Psychiatry and Ageing Research, 14195 Berlin, Germany, and London, UK; Danish Research Centre for Magnetic Resonance, Centre for Functional and Diagnostic Imaging and Research, Copenhagen University Hospital - Amager and Hvidovre, 2650 Copenhagen, Denmark; Vitas AS, Science Park, 0349 Oslo, Norway; Department of Nutrition, Faculty of Medicine, Institute of Basic Medical Sciences, University of Oslo, 0317 Oslo Norway; Center for Lifespan Psychology, Max Planck Institute for Human Development, 14195 Berlin, Germany; Department of Psychiatry, University of Oxford, OX3 7JX Oxford, UK; Faculty of Psychology and Educational Sciences, University of Geneva, 1204 Geneva, Switzerland; UniDistance Suisse, 3900 Brig, Switzerland; Swiss National Centre of Competence in Research LIVES, University of Geneva, 1204 Geneva, Switzerland; Medical Research Council Cognition and Brain Sciences Unit, Department of Psychiatry, University of Cambridge, Cambridge CB2 7EF, England; Department of Psychiatry, University of Oxford, OX3 7JX Oxford, UK; Wellcome Centre for Integrative Neuroimaging, Oxford Centre for Human Brain Activity, University of Oxford, OX3 9DU Oxford, UK; Cognitive Neuroscience Department, Donders Institute for Brain, Cognition and Behavior, Radboud University Medical Center, 6525 GD Nijmegen, The Netherlands; Medical Research Council Cognition and Brain Sciences Unit, Department of Psychiatry, University of Cambridge, Cambridge CB2 7EF, England; Lise Meitner Group for Environmental Neuroscience, Max Planck Institute for Human Development & Clinic for Psychiatry and Psychotherapy, University Medical Center Hamburg-Eppendorf, 20246 Hamburg, Germany; Center for Lifespan Psychology, Max Planck Institute for Human Development, 14195 Berlin, Germany; Max Plank UCL Centre for Computational Psychiatry and Ageing Research, 14195 Berlin, Germany, and London, UK; Danish Research Centre for Magnetic Resonance, Centre for Functional and Diagnostic Imaging and Research, Copenhagen University Hospital - Amager and Hvidovre, 2650 Copenhagen, Denmark; Department of Integrative Medical Biology, Umeå University, 901 87 Umeå, Sweden; Umeå Center for Functional Brain Imaging, Umeå University, 901 87 Umeå, Sweden; Center for Lifespan Changes in Brain and Cognition, University of Oslo, Department of Psychology, University of Oslo, 0373 Oslo, Norway; Danish Research Centre for Magnetic Resonance, Centre for Functional and Diagnostic Imaging and Research, Copenhagen University Hospital - Amager and Hvidovre, 2650 Copenhagen, Denmark; Radiography, Department of Technology, University College Copenhagen, 2200 Copenhagen N, Denmark; Department of Medicine, Faculty of Medicine and Health Sciences, Institut de Neurociències, Universitat de Barcelona, and Institut d’Investigacions Biomèdiques August Pi I Sunyer (IDIBAPS), 08036 Barcelona, Spain; Lübeck Interdisciplinary Platform for Genome Analytics (LIGA), University of Lübeck, 23562 Lübeck, Germany; Department of Psychiatry, University of Oxford, OX3 7JX Oxford, UK; Cognitive Neuroscience Department, Donders Institute for Brain, Cognition and Behavior, Radboud University Medical Center, 6525 GD Nijmegen, The Netherlands; Department of Psychiatry, University of Oxford, OX3 7JX Oxford, UK; Cognitive Neuroscience Department, Donders Institute for Brain, Cognition and Behavior, Radboud University Medical Center, 6525 GD Nijmegen, The Netherlands; Center for Lifespan Changes in Brain and Cognition, University of Oslo, Department of Psychology, University of Oslo, 0373 Oslo, Norway; Center for Computational Radiology and Artificial Intelligence, Oslo University Hospital, 0373 Oslo, Norway; Center for Lifespan Changes in Brain and Cognition, University of Oslo, Department of Psychology, University of Oslo, 0373 Oslo, Norway; Center for Computational Radiology and Artificial Intelligence, Oslo University Hospital, 0373 Oslo, Norway

**Keywords:** aging, individual differences, caudate, hippocampus, cortex

## Abstract

It is well documented that some brain regions, such as association cortices, caudate, and hippocampus, are particularly prone to age-related atrophy, but it has been hypothesized that there are individual differences in atrophy profiles. Here, we document heterogeneity in regional-atrophy patterns using latent-profile analysis of 1,482 longitudinal magnetic resonance imaging observations. The results supported a 2-group solution reflecting differences in atrophy rates in cortical regions and hippocampus along with comparable caudate atrophy. The higher-atrophy group had the most marked atrophy in hippocampus and also lower episodic memory, and their normal caudate atrophy rate was accompanied by larger baseline volumes. Our findings support and refine models of heterogeneity in brain aging and suggest distinct mechanisms of atrophy in striatal versus hippocampal-cortical systems.

## Introduction

In the era of precision medicine, it has been argued that a “one size fits all” approach to cognitive aging is inadequate (Ryan et al. 2019). The same is likely true for brain aging, as some older individuals display relative maintenance of brain structure and function, whereas others show marked age-related changes (Nyberg et al. 2012; Patel et al. 2022). It is well documented that some brain regions are more prone than others to show atrophy, notably caudate, hippocampus, association cortices (e.g. Raz et al. 2005), but the differences in mean change across regions does not preclude individual differences in profiles of change. Specifically, it has been proposed that some individuals display age-related changes in frontal-striatal regions, including the caudate, whereas others show more pronounced changes in hippocampus and in posterior cortical regions, including the precuneus (Buckner 2004). This view is indirectly supported by findings that the caudate and hippocampus form different developmental patterns in relation to cortical maturation (Walhovd et al. 2015) and by suggestions that the hippocampus and caudate have an antagonistic functional and structural relationship (Poldrack and Packard 2003; Sodums and Bohbot 2020).

More direct evidence for heterogeneity comes from studies exploring different “dimensions” of brain aging (related terms include “modes,” “patterns,” “factors,” and "facets"). A factor analysis of data from 317 participants with amnestic mild cognitive impairment (aMCI) in the “Alzheimer’s Disease Neuroimaging Initiative” (ADNI) provided support for multiple change factors, including prefrontal and medial temporal cortex (MTC), and atrophy in these factors was considerably greater in those who converted from aMCI to AD (Carmichael et al. 2013). Eavani et al. (2018) applied multivariate pattern analysis techniques on data from the Baltimore Longitudinal Study of Aging to reveal heterogeneity in structural and functional changes in advanced brain aging. Their findings differentiated resilient and advanced brain agers and suggested distinct subgroups among advanced brain agers, including 1 with elevated focal hippocampal atrophy. A final example comes from analyses of longitudinal data from the Lothian Birth Cohort that revealed 3 dimensions of cortical brain aging (Cox et al. 2021). The major dimension was cortex-wide atrophy that predicted cognitive decline, with a pronounced contribution of dorsolateral prefrontal cortex (DLPFC) and lateral temporal regions, and the other 2 dimensions involved MTC and medial parietal (precuneus) regions, respectively. These and other findings (Smith et al. 2020; Cox and Deary 2022) support heterogeneity in brain aging but do not provide conclusive evidence for individual differences in how aging influences caudate and hippocampus integrity in relation to cortical change (cf., Buckner 2004).

The aim of the present study was to explore heterogeneity in patterns of longitudinal structural brain aging using latent-profile analysis (LPA), a data-driven statistical approach that is suitable for identifying subgroups of individuals within a sample (Masyn 2013; cf., Lövdén et al. 2018). We focused on gray-matter atrophy in the caudate and hippocampus and also in several cortical regions (frontal cortex, lateral cortex, MTC, and precuneus), as measured by magnetic resonance imaging (MRI), in a large longitudinal dataset (1,482 observations) from the “Lifebrain” cohort (Walhovd et al. 2018). Identified subgroups were compared on age, sex, and education as well as “APOE”-distribution and episodic memory. Elevated cortical and hippocampus atrophy was expected to be associated with lower episodic memory and “APOE-ε4” carriership (cf., Buckner 2004; Rast et al. 2018; Cox et al. 2021). For a smaller subset of individuals, the subgroup differences on epigenetic aging profiles were assessed (Horvath 2013) in terms of deoxyribonucleic acid (DNA) methylation (DNAm). Given that DNAm age and age acceleration are considered to represent markers of “biological aging,” we hypothesized a relation between higher epigenetic age acceleration and higher atrophy.

## Materials and methods

### Participants

All participants gave written informed consent. All procedures were approved by a relevant ethics review board. For the Lifebrain consortium, approval was given by the Regional Ethical Committee for South Norway, and all substudies were approved by the relevant national review boards. The sample consisted of 741 healthy (absent of neurological/health problems) participants (baseline age range = 50.5–85.4 years), with 2 observations (mean scan interval = 3.4 y; range ≅ 1–10 years). They were from different studies and European geographical sites (Barcelona, Spain [*n* = 51, *M* age = 69 years]; Berlin, Germany [*n* = 253, *M* age = 70.1 years]; Oslo, Norway [*n* = 156, *M* age = 63.3 years]; and Umeå, Sweden [*n* = 281, *M* age = 65.9 years]) and were aggregated within the Lifebrain project (Walhovd et al. 2018).

### MRI methods and region of interest definitions

The MRI data originated from 6 different scanners and were processed with longitudinal FreeSurfer (version 7.0; https://surfer.nmr.mgh.harvard.edu/) pipeline (Reuter et al. 2012) on the same computer at the University of Oslo. Very noisy images were removed before processing. Identical procedures were used across all samples. Because FreeSurfer is almost fully automated, to avoid introducing possible site-specific biases, gross quality control measures were imposed, and no manual editing was done. As part of the Lifebrain project, 7 volunteers were scanned on 7 scanners in the project (Fjell et al. 2020). Previous comparisons (Fjell et al. 2020) of hippocampal volume showed that the between-participant rank order was almost perfectly retained among scanners (*r* = 0.98). For sites used in the present paper, we ran correlations between the volumetric estimates in each of the regions of interest (ROIs) across sites. This analysis showed correlations close to 1 for all regions except MTL, where correlations were somewhat lower but still >*r* = 0.75.

Bilateral Freesurfer-derived volumes of the Desikan-Killiany atlas were extracted for the ROIs and were then averaged across the right and left hemispheres before being entered into the LPA. The following “FreeSurfer” ROIs were used: (i) hippocampus, (ii) medial temporal lobe (MTL; entorhinal and parahippocampal cortexes), (iii) precuneus, (iv) lateral temporal lobe (LTL; “superior temporal”), (v) caudate nucleus, and (vi) DLPFC (“caudal middle frontal”).

For each ROI and participant, a slope was calculated as %-annual change of volume. To test the potential influence of enlarged ventricles on caudate segmentation accuracy, lateral and inferior lateral ventricle volumes were also extracted and correlated with the caudate volume to investigate possible correlation differences between the groups using the Fischer *r*-to-*z* transformation (Snedecor & Cochran 1980). Brain volume in relation to estimated intracranial volume (eICV) was used as baseline point estimations of atrophy (volume/ICV).

### Episodic memory, APOE-status, DNAm profiling, and education

As previously described (Gorbach et al. 2020), each participating study contributed 1 or several episodic-memory measures. An episodic-memory score for each study was defined by scaling scores at both time points for each individual test by the mean and standard deviation (SD) of the respective test at baseline. Scaling was performed separately for each study due to different scales across sites. A mean over the individual tests within each site was used as episodic memory score. Annual episodic-memory change was also calculated. Within each study, “APOE” allele “ε4” carriership was defined (“ε4”-positive or -negative) by determining the C-allele carrier status at single nucleotide polymorphism rs429358 using targeted genotyping or genome-wide methods analogous to those described in (Sommerer et al. 2022). DNAm profiling (Horvath 2013) was performed using the Infinium MethylationEPIC array (Illumina, Inc) and was processed with the same workflows described previously (Sommerer et al. 2022). Education was quantified as self-reported years of formal schooling, as previously described (Nyberg et al. 2021). Data for these variables used to characterize subgroups were only available for some participants, resulting in different number of participants in the analyses (*N* memory = 714 (at W1), *N* education = 548, “N APOE”-data = 643, and *N* DNAm = 96). Matlab R2015b (Mathworks Inc) was used for data preparation.

### Statistical analyses

LPA was performed using the R software with the tidyLPA package (Rosenberg et al. 2018. Technically, the tidyLPA is a “wrapper” for the mclust package (Scrucca et al. 2016). We assumed that the ROI slopes followed a multivariate Gaussian (normal) distribution in each group and let the respective mean vectors and covariance matrices vary freely between groups. We investigated models using up to 6 groups and used the Bayesian information criterion (BIC) for selecting the number of groups based on results by Tein et al. (2013), showing that, in an LPA setting selection, using BIC has favorable properties over e.g. AIC and entropy measures in selecting the correct number of classes. Tein et al. (2013) showed that the group selection properties of BIC and variants likelihood ratio tests are quite similar. We further modeled ROI volume as a smooth function of age using a generalized additive mixed model (Wood 2017).

After determining the number of classes, interpretation of the outcome was based on inspecting the sizes and means of the latent classes, supplemented by post hoc tests of pairwise group differences in atrophy rates for the ROIs. The purpose of these comparisons was to externally validate and to aid in the interpretation of the identified LPA profiles and to examine if differences remained after controlling for potential confounding factors (age, sex, and site of study). The post hoc nature of these comparisons should be considered when assessing the levels of significance for significant group differences. Between-group differences in episodic-memory change and educational attainment were investigated with pairwise *t*-tests, and the proportion e4-positive versus e4-negative individuals in each class was compared using a chi-square test.

Epigenetic (DNAm) age acceleration was quantified as the residual of a linear regression analysis of chronological age versus DNAm age as described previously (Sommerer et al. 2022). To assess differences in epigenetic age acceleration between identified groups in the LPA, we fit a logistic regression model using group membership as outcome and epigenetic age acceleration, laboratory batch, the first 4 DNAm principal components (PCs), and sex as predictors. DNAm PCs were calculated based on the normalized DNAm beta values and account for the technical variability in the data, as described previously (Sommerer et al. 2022).

### Data availability

The “Lifebrain” data supporting the current results can be requested from the principal investigator of each study, given appropriate ethical and data protection approvals and data transfer agreements.

## Results

Table 1 shows the relation among the analyzed brain measures eICV. Volumes at baseline (mean *r* = 0.42) and volume changes (mean *r* = 0.30) were related, whereas the baseline-change correlations were close to 0 (range = −0.09 to 0.14). Latent profile models specifying one to six classes revealed that a 1-class solution yielded poorer fit to data (i.e. higher BIC value) than all multiclass models. Of the multiclass models, the lowest BIC value was obtained for a 2-class solution, with a margin of 29 to the second-lowest 3-class solution. Using the criteria outlined by Kass and Raftery (1995), a difference >10 is considered as strong evidence in favor of the model with lower BIC. Further investigation using the bootstrapped likelihood ratio test indicated that up to 3 classes were relevant. As the BIC favored 2 classes, we proceeded with the 2-class solution. All study sites contributed to both groups (*N*-group1/*N*-group2): Germany:116/137, Spain:30/21, Norway 104/52, and Sweden:236/45.

**Table 1:** Correlations among regional volumes at baseline (upper) and among regional changes (lower). Baseline volume-change correlations are in bold font at the diagonal.

		Baseline volume						
		DLPFC	Hippocampus	Precuneus	LTL	MTL	Caudate	eICV
Volume change	DLPFC	**−0.09**	0.32	0.52	0.47	0.27	0.31	0.00
	Hippocampus	0.13	**0.14**	0.47	0.48	0.48	0.31	0.07
	Precuneus	0.73	0.17	**−0.04**	0.60	0.39	0.42	0.02
	LTL	0.51	0.26	0.57	**0.00**	0.45	0.48	0.09
	MTL	0.16	0.29	0.19	0.35	**0.01**	0.37	0.03
	Caudate eICV	0.20 0.45	0.15 0.31	0.24 0.60	0.33 0.60	0.15 0.39	**0.01** 0.48	0.00 —

**Fig. 1:**
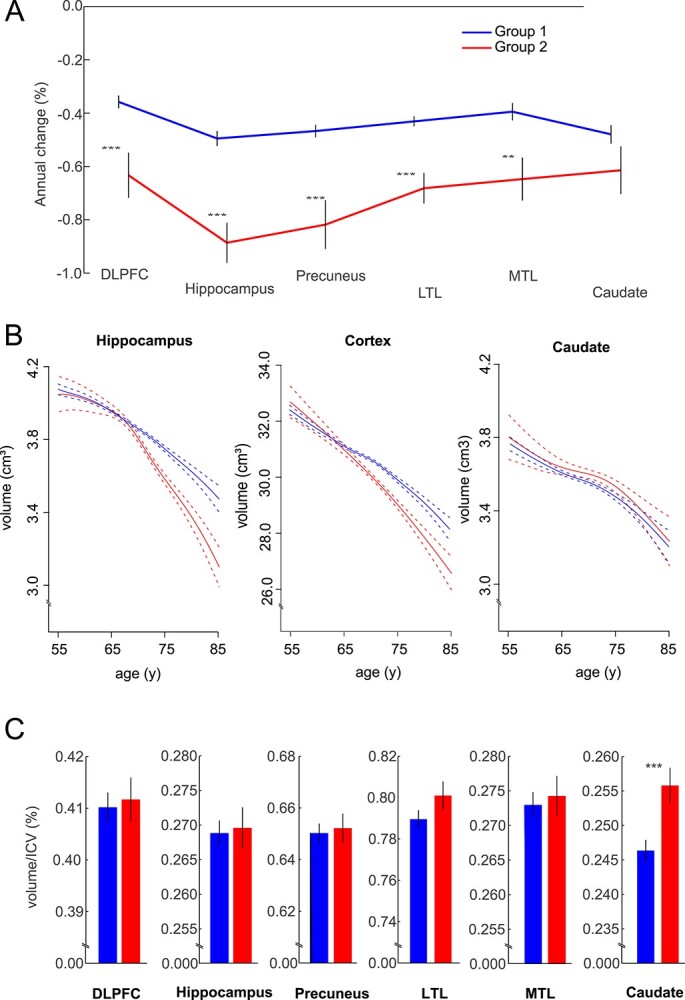
(A) Mean (SE) profiles of atrophy for the 2-class solution. (B) Longitudinal volume changes in hippocampus, cortex, and caudate as a function of age and group (the dotted lines represent 95% confidence intervals). (C) Mean (SE) absolute regional volumes for each ROI and subgroup at the first test wave (W1). ^*^^*^^*^ = *P* < 0.001; ^*^^*^ = *P* < 0.005.

Figure 1A presents the latent profiles of mean atrophy rates in each region for the 2 subgroups. Group 1 (*n* = 486, 66% of the sample, mean age = 66.0 years) showed a relatively homogenous profile of atrophy across all examined areas (amounting to ca 4–5% per decade). Group 2 (*n* = 255, 34% of the sample, mean age = 68.9 years) showed greater cortical and hippocampal atrophy (ranging between ca 6–9% per decade). ANCOVAs (*df* = 734), controlling for age, sex, and study site, confirmed significant group differences in atrophy rates in all cortical regions and hippocampus (*P*-values ranging between 0.0019 and <0.00001). By contrast, caudate atrophy was not significantly different between groups (*P* > 0.05). The latent profile was regionally more heterogenous in group 2, with the greatest atrophy rate for the hippocampus, and hippocampal atrophy was significantly greater than caudate atrophy (*t*(254) = 2.65, *P* = 0.0086), whereas no such difference was seen in group 1 (*t* < 1). In a control analysis, it was found that the correlation between ventricle volume and caudate volume did not differ between groups at baseline (*P* = 0.16) or follow-up (*P* = 0.62).

Figure 1B depicts longitudinal change as a function of age in hippocampus, cortex (across ROIs), and caudate for the 2 groups. These plots confirm more rapid atrophy for group 2 in cortex and hippocampus after age 65, whereas the caudate volume changed in a similar fashion in both groups. The change function estimates indicated greater caudate volume in group 2 than in group 1 across age (Fig. 1B; red > blue solid line). To directly compare regional levels (i.e. intercepts) between the groups, for each individual, we computed the volume of each region at study onset in relation to ICV. Despite being older, group 2 had a significantly larger baseline caudate volume than group 1 (*t*(739) = −3.32, *P* = 0.00094; Fig. 1C). No other ROI differences were significant, and a comparison of total brain volume in relation to eICV revealed no significant difference between the 2 groups at the baseline (*t* = −1.8, *df* = 737, *P* = 0.072) or in change between test waves (*t* = −0.15, *df* = 737, *P* = 0.88).

Next, we conducted additional analyses to characterize the identified groups (Table 2). The groups did not differ with regard to education (*t*(546) = −0.64, *P* = 0.52), APOEε4-carriership (χ^2^(1) = 1.44, *P* = 0.23), but group 2 was older (*t*(739) = −6.02, *P* < 0.001) and included more men (χ^2^(1) = 4.48, *P* = 0.034]. For episodic memory, the annual change was greater in group 1 (*t*(679) = 2.24, *P* = 0.025), but their performance level was still greater than that of group 2 at both the first (*t*(712) = 4.10, *P* < 0.001] and second test waves (*t*(703) = 2.36, *P* = 0.018).

**Table 2:** Group characteristics.

Variable	Group 1	Group 2
*N* (female/male in %) Mean baseline age (range) Education (*M* in years) Episodic memory (*M*, SD)^a^ APOE (% ε4)	486 (48/52) 66.0 (50.5–82.7) 13.9 0.076 (0.75), −0.031 (0.78) 27	255 (40/60) 68.9 (50.6–85.4) 14.1 −0.17 (0.78), −0.19 (0.99) 22

^a^Values for the first and second test waves.

Last, we assessed whether the 2 groups differed with respect to their epigenetic aging profiles. Specifically, we computed the degree of epigenetic age acceleration for each individual with overlapping MRI and DNAm data (*n*_group 1_ = 47, *n*_group 2_ = 49) and assessed whether the 2 groups showed differences in epigenetic age acceleration. The results of these analyses did not indicate different epigenetic age acceleration between the groups (beta = 0.0032, *P*-value = 0.92), but they were most likely underpowered.

## Discussion

Using latent profiling on longitudinal volumetric brain-atrophy data, we obtained support for the existence of 2 subgroups of individuals within our sample, which differed in their atrophy profiles. The first group included about two-thirds of the sample and had a fairly uniform pattern of atrophy across all 6 examined cortical and subcortical ROIs, with an annual shrinkage rate of about 0.5% which is comparable with the previous estimates of mean atrophy rates across individuals (Fjell et al. 2009). The change pattern was approximately linear, which agrees with past observations for these regions across the age span investigated here (Sørensen et al. 2021). The second subgroup showed higher atrophy rates in the cortex and hippocampus. The higher-atrophy group was slightly older, and the variation in age across the included cohorts may explain the uneven distribution of participants between groups (the German sample had the highest percentage of participants in the high-atrophy group and also the highest mean age). Also, the higher-atrophy group included more males relative to females, but the group differences in atrophy rates remained after controlling for both age and sex.

The groups were comparable with regard to APOE-distribution, education, and epigenetic age (for the latter variable, a limitation is that the analyses were underpowered). The higher-atrophy group had lower episodic memory at both waves, which is consistent with prior reports of a link between poorer memory and hippocampal atrophy (Gorbach et al. 2020; Johansson et al. 2022). In the higher-atrophy group, the lower initial performance level together with less annual change in memory decline might have reflected a pathological cascade that was initiated well before the baseline session.

In the higher atrophy group, marked hippocampal atrophy was accompanied by elevated atrophy in all examined cortical ROIs. Thus, although the greatest cortical atrophy numerically was seen in the precuneus, no support was found for selective cortical atrophy (Buckner 2004). Rather, the broad pattern of cortical atrophy resembles findings from the ADNI (Carmichael et al. 2013) and the major change dimension in the Lothian birth cohort (Cox et al. 2021). In view of the broad pattern of atrophy, it remains possible that other cognitive processes, in addition to episodic memory, were affected. Indeed, there is recent evidence that adult cognitive decline or maintenance show a strong dependency among various measures of fluid and crystallized ability (Tucker-Drob et al. 2022).

Strikingly, the rate of caudate atrophy did not differ between the groups. Within the higher-atrophy group, we also found that hippocampus atrophy was significantly greater than the caudate atrophy. These observations are in line with the suggestion that the hippocampus and striatum can follow different trajectories during aging (Gardner et al. 2020). Moreover, despite being older, individuals in the higher-atrophy group had larger caudate baseline volumes, whereas the volumes of other regions (and total brain volume) were comparable between groups. It remains unclear whether larger caudate volumes contributed to normal atrophy rates in the second group, but it has been proposed that smaller hippocampal volumes in aging will bias toward striatal computation and functional brain activity (e.g. Konishi et al. 2013; cf. Bohbot et al. 2007, 2012; Schuck et al. 2015; Sodums and Bohbot 2020) and that larger caudate volumes could reflect a compensatory response to reduced hippocampus-driven function (Persson et al. 2017).

The observed heterogeneity in atrophy profiles may relate to evidence that the genetic influences on atrophy rates differ for cortex, hippocampus, and the caudate (Fjell et al. 2021; Brouwer et al. 2022). In the Brouwer et al. study, genes involved in interacting with the tau protein were associated with the rate of change in caudate volume. Aggregation of tau in aging and neurodegeneration also contributes to elevated cortical and hippocampal atrophy (La Joie et al. 2020). Additional genes with distinct influences on the caudate relative to hippocampus and cortex likely also play a role (Fjell et al. 2021).

Future studies will be needed to replicate these findings and to address various limitations. We cannot rule out confounds related to technical issues due to iron-induced bias in volume estimations (Lorio et al. 2014) or to the automatic segmentation of striatal volumes, although the control analyses did not indicate any confounds in relation to ventricle size. We examined a limited set of subcortical and cortical volumes. Although we argue that this selection captured the relevant and aging-sensitive parts of the cerebrum, it cannot be ruled out that the analyses of other or additional regions, possibly with a whole-brain high-dimensional coverage, would yield different outcomes. Also, investigating the cortical thickness or surface area instead of volumes could potentially lead to different outcomes in relation to the magnitude of age changes and cognition (Borgeest et al. 2021). Here, with no a priori hypotheses concerning individual differences in change, we found cortical volumes as a reasonable point of departure that harmonized with the subcortical volumes.

## Conclusion

Taken together, our findings support and refine models of heterogeneity in brain aging by showing that about a third of the participants (i) were characterized by elevated hippocampus atrophy, (ii) had elevated cortical atrophy globally rather than selectively in frontal or posterior cortex, and (iii) showed similar rates of caudate atrophy as the lower-atrophy participants. Additional studies with complementary imaging markers (e.g. tau-PET) will be needed to specify the mechanisms that contribute to heterogeneity in brain aging.
